# Supplementary figures and images for: Validation of Lead-DBS β-Oscillation Localization with Directional Electrodes
Source: Bioengineering (Basel). 2023 Jul 28;10(8):898. doi: 10.3390/bioengineering10080898 (PMC10451384; doi:10.3390/bioengineering10080898)

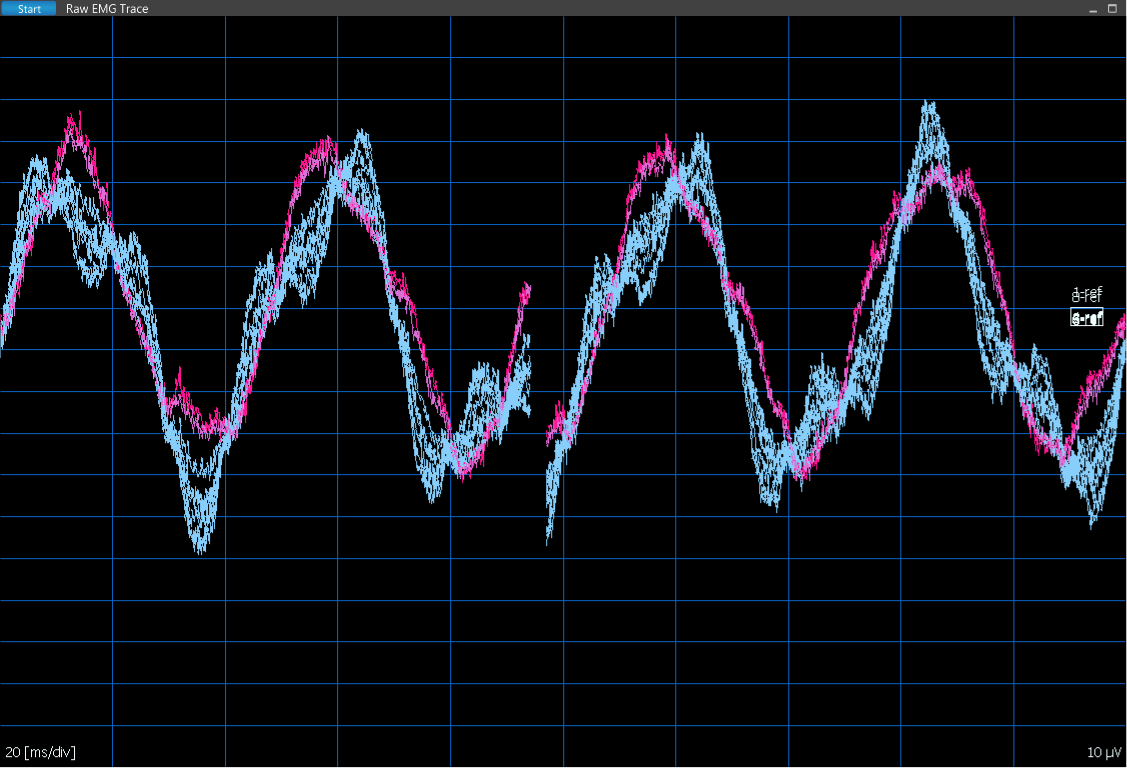

Supplement: Supplementary file 1 [file bioengineering-10-00898-s001.zip › Supplementary Figure S1 (21Hz).PNG]

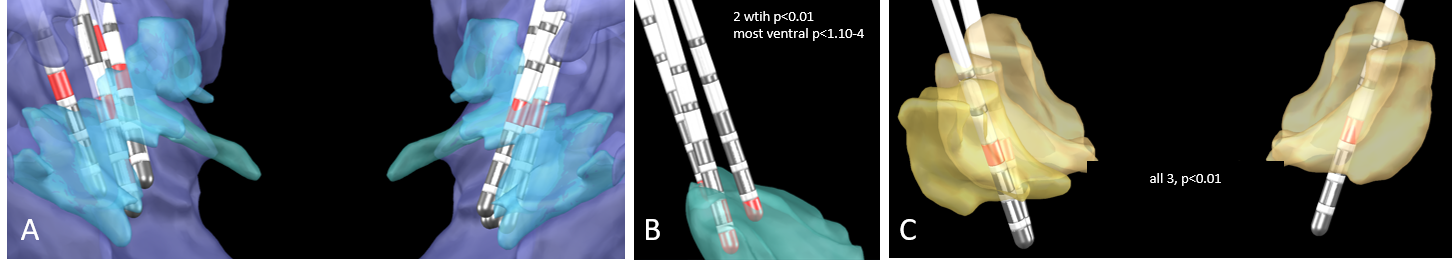

Supplement: Supplementary file 1 [file bioengineering-10-00898-s001.zip › Supplementary Figure S2 Other sites of Beta.tif]
